# Aortic Thickness: A Forgotten Paradigm in Risk Stratification of Aortic Disease

**DOI:** 10.1055/s-0040-1715609

**Published:** 2020-12-23

**Authors:** Ashutosh Hardikar, Robin Harle, Thomas H. Marwick

**Affiliations:** 1 Menzies Institute for Medical Research, University of Tasmania, Australia; 2 Department of Cardiothoracic Surgery, Royal Hobart Hospital, Hobart, Australia; 3 Department of Radiology, Royal Hobart Hospital, Hobart, Australia; 4 Baker Heart and Diabetes Institute, Melbourne, Australia

**Keywords:** aortic wall thickness, aortic dilatation, aortopathy, aortic stress

## Abstract

**Background** This study aimed at risk-stratifying aortic dilatation using aortic wall thickness (AWT) and comparing methods of AWT assessment.

**Methods** Demographic, epidemiological, and perioperative data on 72 consecutive aortic surgeries (age = 62 years[standard deviation (SD) = 12] years) performed by a single surgeon were collected from hospital database. Aortic thickness was measured on computed tomography scans, as well as intraoperatively in four quadrants, at the level of aortic sinuses, as well as midascending aorta, using calipers. Aortic wall stress was calculated using standard mathematical formulae.

**Results** The ascending aorta was 48.2 (SD = 8) mm and the mean thickness at ascending aorta level was 1.9 (SD = 0.3) mm. There was congruence between imaging and intraoperative measurements of thickness, as well as between the radiologist and surgeon. Preoperatively, 16 patients had multiple imaging studies showing an average rate of growth of 1.2 mm per year without significant difference in thickness. The wider the aorta, the thinner was the lateral or convex wall. Aortic stenosis ( *p* = 0.01), lateral to medial wall thickness ratio ( *p* = 0.04), and history of hypertension ( *p* = 0.00), all had protective effect on aortic root stress. The ascending aortic stress was directly affected by age ( *p* = 0.03) and inversely related to lateral to medial wall thickness ratio ( *p* = 0.03).

**Conclusion** Aortic thickness can be measured preoperatively and easily confirmed intraoperatively. Risk stratification based on both aortic thickness and diameter (stress calculations) would better predict acute aortic events in dilated aortas and define aortic resection criteria more objectively.

## Introduction

The aortic dilatation associated with bicuspid aortic valves (BAV), pathologic tricuspid aortic valves (TAV), and other aortopathies is clinically relevant due to the risk of acute aortic events. Risks for acute aortic events are governed by genetic predisposition, hemodynamic factors, and morphology. The law of Laplace (Duprey et al 1 ), best explained by the Young–Laplace equation, shows wall stress to relate to the internal pressure ( *P* ), radius of the aneurysm ( *R* ), and aortic wall thickness (AWT;“ *t* ”).

Hypertension, wall thinning, and aortic enlargement are the most important factors increasing aortic wall stress and leading to aortic rupture or dissection. 2 Aortic diameter has been the primary measurement in the literature, 3 and guidelines have concentrated only on aortic diameters. 4 Few studies have focused on blood pressure, 5 and very few have considered aortic thickness. 6 Some surgeons have used subjective criteria of friability or tissue quality, or indexing body size to facilitate intraoperative decisionmaking in aortas <45 mm. 7 Some studies have highlighted the possibly catastrophic thinness of aortas. 8 We here present an easy method of aortic thickness measurement and wall stress calculation to objectively prognosticate the risk of future aortic events.

## Materials and Methods

### Study Design

The study was approved under aortic pathology registry by the Tasmanian Human Research and Ethics committee (Reference no.: H0013456). Individual patient consents were obtained to join the registry. Demographic, epidemiological, and perioperative data were collected from a database at the Royal Hobart Hospital (RHH). AWT measurements were obtained on computed tomography (CT) imaging studies in 72 consecutive aortic surgeries by a single surgeon between 2014 and 2018, and interobserver variability was assessed by a specialist radiologist in 10 randomly selected cases. The intraoperative thickness was measured using calipers in four quadrants at the level of the aortic sinuses, as well as the mid-ascending aorta. The measurement was done on-table before sending the specimen for pathology using standard calipers and using a point where one cannot pull the aortic wall out of the calipers as the thickness. 9 The stress in aortic wall was calculated using standard mathematical formula; aortic wall circumferential stress = ( *P* × *R* ) / *t* ; where *P* is the systolic blood pressure of the aorta while measuring the diameter, *R* is the radius, and *t* is the AWT.

In three patients, thickness could not be measured intraoperatively in all four quadrants (aortic dissection cases). Imaging studies were unable to measure the exact thickness in at least one quadrant in seven cases.

### Epidemiology

Confounding variables which would likely affect aortic thickness including sociodemographic, morphometric (height, weight, and dimensions), hypertension, diabetes, dyslipidemia, vasculopathy, renal disease, smoking history, aortic valve pathology, and medications were entered ( Table 1 ). A family history of aortic or cardiovascular disease was recorded if a parent or a sibling was affected.

**Table 1:** Demographic, clinical, and morphometric characteristics of 72 patients undergoing aortic surgery

Characteristic	Total	Aneurysm	Dissection	*p* -Value
Number	72	58	14	NA
*Demographics* :				
Male ( *n* )	58	49	9	0.12
Age in year (SD)	62.04 (11.6)	61.95 (12.1)	62.40 (9.7)	0.91
*Anthropometric* :				
Height in cm (SD)	173.87 (8.28)	173.53 (8.31)	175.30 (8.42)	0.55
Weight in kg (SD)	83.17 (20.6)	85.12 (17.6)	74.8 (30.3)	0.16
Body surface area in m ^2^ (SD)	1.99 (0.20)	1.99 (0.20)	1.97 (0.22)	0.76
*History* :				
Hypertension	46	35	11	0.20
Chronic obstructive pulmonary disease	11	9	2	0.91
Peripheral vascular disease	5	5	0	0.26
Diabetes	1	1	0	0.62
Dyslipidemia	26	23	3	0.20
Smoker	35	28	7	0.91
*Clinical features* :				
≥ Moderate aortic stenosis	27	27	0	**0.01**
≥ Moderate aortic regurgitation	36	28	8	0.14
Bicuspid aortic valve	31	29	2	**0.02**

Abbreviation: SD, standard deviation.

### Surgery

Aortic resection was performed in accordance with the recent guidelines. 10 The indication was primary pathology meeting size criteria or rapid growth in aneurysm size in 26 cases, concomitant surgery in 32 cases, and acute aortic dissection in 14 cases.

### Intraoperative Measurement

Aortic diameter was measured intraoperatively by measuring the circumference of the aorta and deriving the diameter. Aortic thickness was measured in four quadrants, anterior, posterior, medial, or inner/concave border (toward the pulmonary artery) and lateral or outer/convex border (toward the superior vena cava). Measurements were done at mid-ascending aorta or the aortic root or both, depending on the extent of aortic resection ( Table 2 ). A surgical grade Vernier's caliper was used to measure aortic thickness. A note of type of aortic valve and its pathology was made at the same time.

**Table 2:** Aortic dimensions/measurements at time of surgery

	Total ( *n* = 72)	Aneurysm ( *n* = 58)	Dissection ( *n* = 14)	*p*
Aortic annulus	26.72 (SD = 2.5)	27.20 (SD = 2.4)	24.74 (SD = 1.9)	**0.00**
Aortic root(the sinus of Valsalva)	44.75 (SD = 9.6)	45.10 (SD = 10.1)	43.36 (SD = 7.3)	0.55
Sinotubular junction	40.58 (SD = 9.6)	40.24 (SD = 9.8)	41.95 (SD = 8.6)	0.55
Ascending aorta	48.19 (SD = 8)	47.45 (SD = 7.7)	51.26 (SD = 8.7)	0.11
Proximal arch	33.68 (SD = 6.7)	33.65 (SD = 6.9)	33.82 (SD = 5.9)	0.93
Left ventricular outflow tract–root angle	12.38 (SD = 5.6)	11.89 (SD = 4.6)	14.40 (SD = 8.3)	0.13
Root–ascending aorta angle	30.27 (SD = 12.2)	29.75 (SD = 12.9)	32.43 (SD = 8.9)	0.46
Aortic valve angle	46.80 (SD = 8.5)	47.15 (SD = 8.8)	45.38 (SD = 7.2)	0.49
Asymmetry index	1.09 (SD = 0.1)	1.09 (SD = 0.1)	1.09 (SD = 0.1)	0.92
Root thickness (mm)	1.88 (SD = 0.36)	1.84 (SD = 0.32)	2.18 (SD = 0.51)	0.05
Ascending aorta thickness (mm)	1.91 (SD = 0.30)	1.88 (SD = 0.28)	2.02 (SD = 0.39)	0.11
Lateral/medial wall ratio aortic root	0.86 (SD = 0.1)	0.86 (SD = 0.1)	0.85 (SD = 0.1)	0.73
Lateral/medial wall ratio ascending aorta	0.86 (SD = 0.1)	0.86 (SD = 0.1)	0.87 (SD = 0.1)	0.60
Aorta root stress (mm Hg)	1,486.72 (SD = 475)	1,529.8 (SD = 476)	1,211.3 (SD = 398)	0.94
Aortic stress (mm Hg)	1,402.68 (SD = 314)	1,404.33 (SD = 331)	1,396.53 (SD = 252)	0.17

Abbreviation: SD, standard deviation.

Note: Values are expressed as mean and standard deviation.

### Imaging

**Fig. 1:**
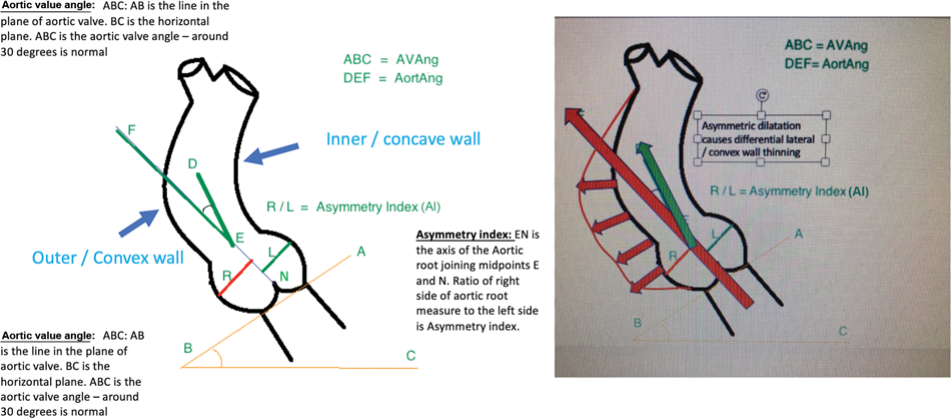
Aortic angles and asymmetry index.

All 72 patients had a preoperative imaging studies performed in a standard protocol and 21 had more than one study before surgery. Aortic diameters were measured at predetermined levels, and asymmetry index (AI) was derived ( Fig. 1 ). Aortic thickness measurements were assessed in four quadrants at the aortic root and mid-ascending aorta level ( Table 2 ). Three different angles, namely, aortic valve angle (AVAng), left ventricular outflow tract (LVOT)–root angle (RootAng), and root-aorta angle (AortAng) were measured ( Fig. 1 ).

### Interobserver Variability

In a random selection of 10 representative studies, all measurements were obtained twice by primary investigator (A.H.) on separate occasions and repeated by an experienced radiologist (R.H.). The mean measurement was taken and percentage deviation from the mean was used to estimate measurement variability.

### Statistical Analysis

The statistical analysis was performed using a standard statistical software package (IBM Corp. Released 2017. IBM SPSS Statistics for Macintosh, Version 23.0. Armonk, NY; IBM Corp.). Continuous data are presented as mean (standard deviation [SD]). Correlations among different variables were performed using Pearson's or Spearman's test where appropriate. We also performed univariable and multivariable analyses for the study of association of aortic stress with different parameters. The standardized beta values with confidence intervals and probability values are presented. The inter- and intraobserver agreement for the aortic root measurements was described by kappa statistics. Reproducibility was assessed using Bland–Altman analysis and intraclass correlation coefficient (ICC). We considered an *α* < 0.05 to be significant.

## Results

### Patient Characteristics

The demographic, morphometric parameters along with other variables affecting aortic thickness are given in Table 1 . The aneurysm and dissection groups were essentially similar except that dissection group had more proportion of females and hypertensive patients (not statistically significant), while aneurysm group patients were more likely to have a bicuspid aortic valve or more than moderate aortic stenosis ( *p* ≤  0.02).

### Aortic Wall Thickness

**Fig. 2:**
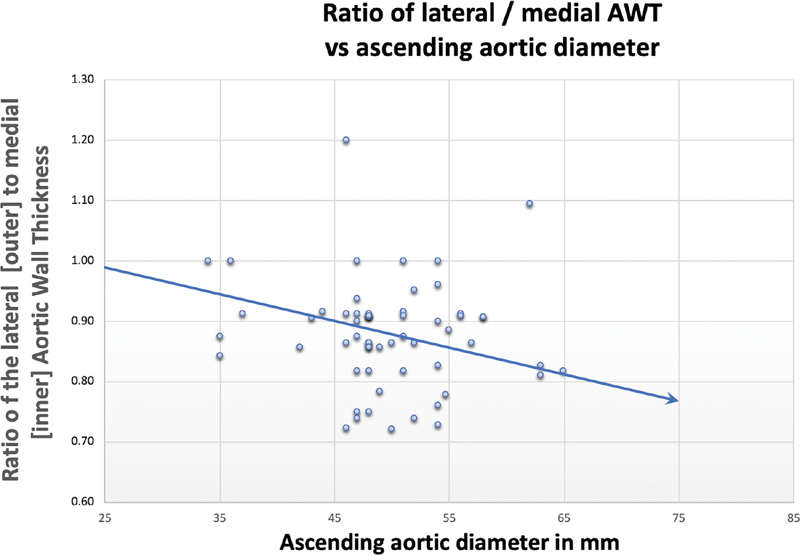
Relationship of convex/outer to concave/inner wall thickness ratio to aortic diameter in the ascending aorta. Lateral to medial aortic wall thickness ratio reduces as the aorta dilates.

The mean thickness in four quadrants of the aortic root, mid-ascending aorta, or both, along with aortic diameters from annulus, sinuses, sinotubular junction, mid-ascending aorta, and different arch and descending levels along with aortic angles are given in Table 2 . Average aortic root thickness was different in aneurysm (1.84 (SD = 0.3] mm) and dissection (2.18 [SD = 0.5] mm) groups ( *p* = 0.05). The aortic annuls was also wider in the aneurysm group (27.2 [SD = 2.4] mm) versus the dissection group (24.7 [SD = 1.9] mm). We plotted a ratio of outer/convex to inner/concave wall thickness against the aortic diameter at the level of ascending aorta ( Fig. 2 ), which shows that the ratio reduces as the aorta dilates, indicating a preferential thinning of the convexity of the aorta.

### Aortic Wall Stress

Table 3 shows the multivariable regression analysis for aortic wall circumferential stress, derived from operative measurements. Ratio of outer/convex to inner/concave AWT significantly affected the aortic stress at both ascending aorta and root levels ( *p* ≤  0.05). Furthermore, age correlated with ascending aortic stress ( *p* ≤  0.05), while hypertension history was associated with lower stress levels in the aortic root ( *p* < 0.001). Aortic stenosis had a significant protective effect on aortic root stress ( *p* = 0.01), while the female sex had some effect as well (beta = 1.83, *p* = 0.08).

**Table 3:** Independent associations of aortic wall stress

For aortic root	Model *R* ^2^ = 0.58, *p* = 0.00		
*Parameter*	Beta (CI)	“ *t* ” value	*p* -Value
Height (cm)	−0.25 (−29.1 to 3.5)	−1.6	0.12
Sex	0.27 (−33.4 to 613.3)	1.83	0.08
Hypertension history	−0.78 (−920.5 to −436)	−5.73	**0.00**
Lateral/medial aortic wall thickness ratio	−0.28 (−2165 to −69.9)	−2.18	**0.04**
Aortic valve angle	0.1 (−7.55 to 17.51)	0.81	0.42
Aortic stenosis	−0.41 (−229 to 36)	−2.81	**0.01**
Aortic regurgitation	0.01 (−70 to 135.6)	0.65	0.52
For ascending aorta	Model *r* ^2^ = 0.28, *p* = 0.08		
*Parameter*	Beta (CI)	“ *t* ” value	*p* -Value
Body surface area	0.19 (−123.2 to 750.7)	1.44	0.16
Age	0.3 ( 1.1 to 17.1)	2.28	**0.03**
Hypertension history	−0.12 (−272.8 to 97.4)	−0.95	0.35
Lateral/medial AWT ratio	−0.35 (−2681 to −121.7)	−2.2	**0.03**
Root–ascending aorta angle	−0.29 (−16.7 to 0.56)	−1.88	0.07
Aortic stenosis	0.0 (−99.5 to 101.9)	0.02	0.98
Aortic regurgitation	−0.0 (−89.3 to 78.2)	−0.02	0.90
Sex	−0.22 (−447.2 to 59.7)	−1.5	0.13

**Fig. 3:**
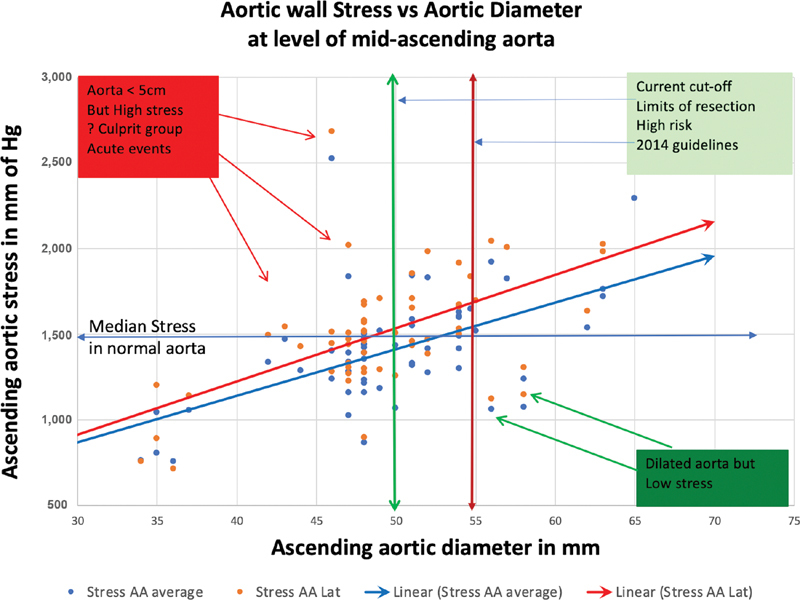
Relationship of aortic wall stress to aortic diameter in the ascending aorta. Wall stress increases as the aorta dilates, but the most meaningful observations may be the outliers. AA, ascending aorta.

Figure 3 shows the association of aortic wall stress with aortic diameter. While wall stress and diameter are concordant in most patients, a meaningful number of patients have high wall stress despite a small diameter, or low wall stress with a high diameter.

**Fig. 4:**
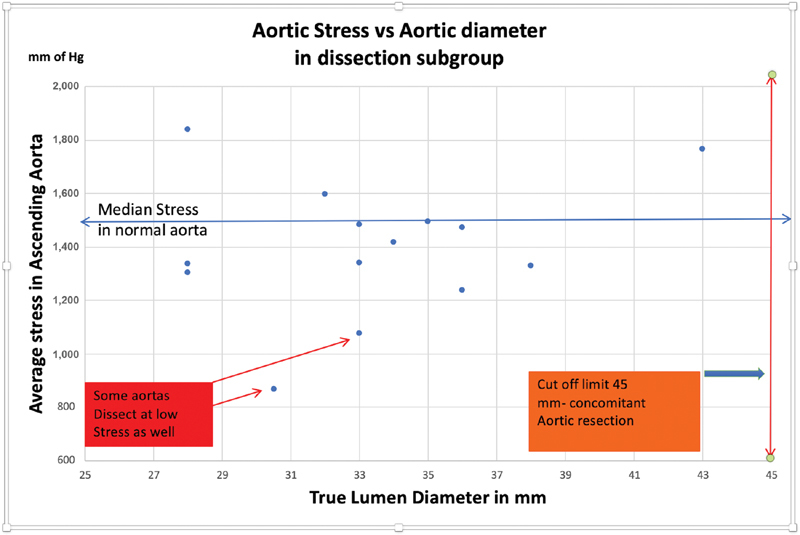
Wall stress versus aortic lumen diameter in 14 patients with aortic dissection. In 13 cases, the true lumen diameter was <45 mm and the wall stress was low, emphasizing the role of tissue characteristics or blood pressure surges.

Figure 4 shows aortic stress plotted against true lumen diameter in 14 patients with aortic dissection. We measured the true lumen based on a previous article by Neri et al. 11 This measurement was done on-table after we excised the dissected segment, before sending it off to the pathologist. Thirteen of these had true lumen diameter (prior to dissection) of <45 mm. In dissected aortas, we measured AWT of the dissected aorta by putting together the layers after removing any blood clot/thrombus in between the layers. We measured thickness at the mid-ascending aortic level (over the main pulmonary artery) for ascending aorta at all times.

### Thickness Measurement over Time

The aortic measurements in 21 patients with more than one preoperative imaging studies, including aortic diameters and thickness showed that average growth in the aortic diameter was 1.19 mm per year, but there was no significant change in the AWT.

### Correlation between Imaging and on-Table Actual Measurements

**Fig. 5:**
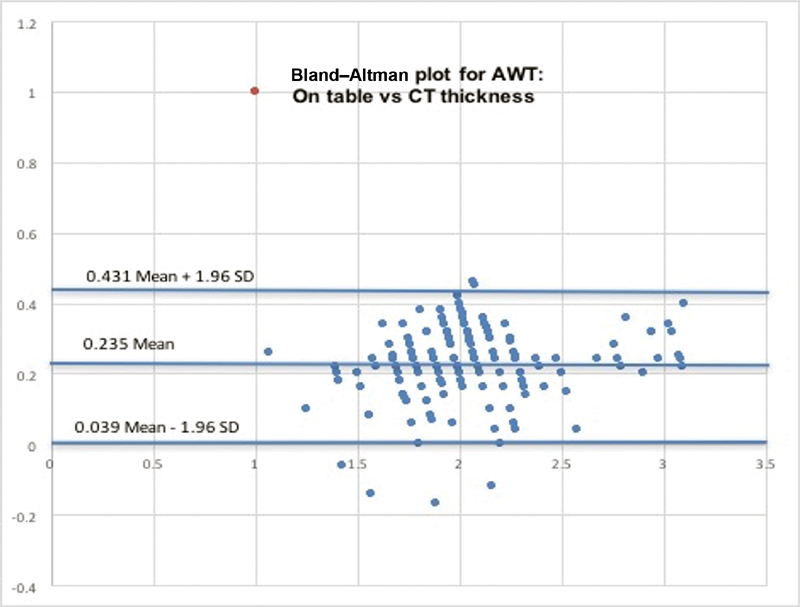
Agreement between intra-operative and computed tomography assessment of aortic wall thickness. AWT, aortic wall thickness; CT, computed tomography; SD, standard deviation.

The kappa values for intraobserver variation was 0.73 while between the surgeon and radiologist, it was 0.62, suggesting substantial agreement. The Bland–Altman plot for comparison of on-table AWT measurement with the CT scan measurements ( Fig. 5 ) had coefficient of 0.18 ( *p* = 0.01), showing a weaker agreement.

## Discussion

These results show that preoperative measures of aortic thickness are possible and readily validated by intraoperative measurements. Risk stratification based on both aortic thickness and diameter would better predict aortic events in patients with dilated aortas.

### Aortic Thickness

Aortic thickness measurements in the normal population 12 show increasing thickness with age, affected by race and gender. AWT and distensibility are related to cardiovascular risk factors, including hypertension. 13 AWT has also been shown to predict lifetime risk of cardiovascular disease in middle-aged individuals with a low short-term risk of coronary heart disease. 14 In addition to age, gender, and ethnicity, AWT is affected by smoking status, systolic blood pressure, high-density lipoprotein (HDL) and low-density lipoprotein (LDL) cholesterol levels, and fasting blood sugar level. 15 Interestingly, a conclusive relation between increased AWT and coronary artery disease has not been shown. 16 The other associations of AWT are with hypothyroidism, 17 increased cerebrovascular events, 18 and possibly for diagnosis of aortitis. 19

AWT is a risk factor in aortic dissection. 20 Previous studies using finite element model have predicted that assessment of regional AWT would better predict the dissection risk. 21 The aortic stress in the dissection subgroup (1,386 [SD = 298] mm Hg) trended to be lower than the aneurysm subgroup (1,448 [SD = 351] mm Hg) which indicates that either blood pressure surges are at work, 22 or the dissected aortas were weaker and there are factors beyond the Law of Laplace like aortic strength or elasticity are relevant. AWT has been measured by epiaortic echocardiography and used to calculate mechanical characteristics of the aorta. 23

### Measurement of Aortic Wall Thickness

Population-based values of AWT are variable, and probably dependent on imaging modality with an echocardiographic AWT of 2.4 (SD = 0.8) mm, 24 compared with a median value of 1.5 mm by magnetic resonance imaging. 25 This variation has been taken into consideration in mathematical models. 26 Due to this variation in assessment of thickness, it has been recommended that intra vascular ultra sound might be the most accurate in measuring AWT. 27 Our measurements of mean ascending aortic thickness of 1.91 mm and aortic root thickness of 1.88 mm are close to that reported in literature.

In the absence of objective measurements or indices, the traditional intraoperative assessment of the quality and the AWT has been subjective. 28 Although magnetic resonance imaging (MRI) was first used to calculate AWT, 12 CT is more widely available and seems equally effective. Semiautomated analysis of CT aortogram images can be used to identify circumferential variations in AWT. 29 Of the echocardiographic methods, the epiaortic probe appears the most robust. In our study, the CT measurement of AWT slightly overestimated the intraoperative measurement. The intra- and interobserver agreement of CT measures are fair.

The measurement of AWT in pathology specimens 20 is unlikely to be accurate. 30 Even when the postmortem CT scan was done only 20 hours after dissection, the AWT increased in the range of 20 to 25%. We have hence used intraoperative caliper measurements in all our calculations, hoping this measure to be closest to the actual AWT in vivo. AWT is variable along the circumference (some studies separately mention the mean and maximal AWT), and during surgery, a surgeon can inspect and sample the thinnest or most friable area and have an objective assessment of the possible stress generated at that spot for a given blood pressure.

We agree that it is possible that the AWT might change once it is excised, as the aortic wall is no longer under the pressure when normally it was in a cylindrical structure with blood in it. However, we believe that, first of all, the change should be minimal as compared with other studies who have done measurements from specimens fixed in formalin. Second, the change would uniformly affect all the specimen, so any variation should be similar across the board.

For the same reason, we possibly have only a weak correlation (Bland–Altman plot coefficient of 0.18) for comparison of preoperative CT image measurement and intraoperative measurement with calipers.

### Contributors to Wall Stress

Hypertension and the strength of the aortic wall may contribute to aortic dilatation in aortopathies. Aortic size increases at more than double the rate in hypertensive than normotensive patients after aortic valve replacement (AVR). 31 However, observations of aortic dilatation after AVR are not uniform; Yasuda et al 32 showed that the BAV group were not affected by hemodynamic parameters, whereas the TAV group had increased dilatation for higher diastolic blood pressure and higher fractional shortening. In our study group, a history of hypertension actually had a protective effect on aortic wall stress. Likewise, although Forsell et al 33 showed decreased wall thickness in BAV as compared with TAV, our study showed similar wall thickness in BAV, as well as TAV. Another factor to note is that only 1 of the 72 patients had diabetes. It is possible that diabetes might affect the aortic stiffness and have a protective effect, 34 although the data are small to draw any conclusions.

Aortic morphology is also important. The RootAng was different between the aneurysm and dissection subgroups (but not statistically significant) suggesting a role for hemodynamic forces in the dissection patients. Fig. 2 illustrates that with increasing aortic diameter, the ratio of outer/convex versus inner/concave wall thickness reduces ( *p* = 0.04), which explains why the convexity of the aorta is more vulnerable to acute aortic events with increasing diameter.

### Clinical Implications

A comprehensive assessment of the aortic wall stress, based on the aortic diameter and the AWT, taking into account whether the patient is hypertensive or not, would yield a more objective assessment about the decision of aortic surgery in a given patient. To some extent, this mirrors practice by some surgeons. Bauer et al 28 have argued that during reduction aortoplasty, either the effective diameter of the aorta should be reduced to <35 mm or the wall should be supported externally with a Dacron graft. This reduces the effective stress in the aortic wall by either reducing the diameter or increasing the effective thickness. The role of wall thickness may explain the 40% of patients in the International Registry of Acute Aortic Dissection (IRAD) registry with aortic dissections despite a diameter <5 cm. 35 Preoperative assessment by ultrasound, CT, or MRI can be used to predict the variation in the AWT, and further on-table measurement of a sample of the aortic wall may help to inform decisions.

## Limitations

The small numbers in this study are a reflection of the study design that provided consistent measurement of thickness by a single surgeon. The data (especially Fig. 4 ) indicates that either significant blood pressure surges cause acute aortic syndromes, 22 or all aortas are not created equal and even the Law of Laplace might not completely explain the risk of acute aortic events. We utilized intraoperative blood pressures for calculating aortic wall stress, which would be obviously much lower in an anaesthetized patient. We have not actually shown that incorporating AWT enhances the prediction of aortic dissection, we are proposing that it might improve the predictive accuracy.

## Conclusion

Using aortic diameter alone to quantify the risk of acute aortic events is proven inadequate. Aortic thickness is an important parameter in the assessment of wall stress that can be measured preoperatively and easily confirmed intraoperatively. Risk stratification, based on calculation of aortic wall stress, might help to individualize decision-making.
